# MRI outperforms CT for tracheal and vascular invasion staging in esophageal cancer

**DOI:** 10.1007/s00330-025-12080-4

**Published:** 2025-10-25

**Authors:** Yumiko Kono, Takashi Harino, Shintaro Yamamoto, Ryo Ogasawara, Kohiro Akita, Makoto Yamasaki, Noboru Tanigawa

**Affiliations:** 1 Department of Radiology, Kansai Medical University, Osaka, Japan; 2 Department of Upper GI Surgery, Kansai Medical University, Osaka, Japan; 3 Department of Radiology, Kansai Medical University Hospital, Osaka, Japan

**Keywords:** Esophageal neoplasms, Magnetic resonance imaging, Neoplasm staging, Trachea, Blood vessels

## Abstract

**Objectives:**

To validate a standardized MRI scoring system, tracheal invasion score (T-score) and vascular invasion score (V-score) against CT for detecting tracheal and major-vessel invasion in esophageal cancer, based on imaging obtained after neoadjuvant therapy.

**Materials and methods:**

Twenty-six patients (mean age 65 years) who underwent both MRI and CT after preoperative therapy and prior to esophagectomy were retrospectively reviewed. Two radiologists independently assigned T- and V-scores on MRI and CT-based T-stage (12th Japanese Classification). Diagnostic performance was measured by the area under the ROC curve (AUC) and *κ* for inter-reader agreement. Patient-level bootstrap resampling (2000 iterations) compared the combined MRI score—defined as max (T, V)—with CT.

**Results:**

MRI yielded higher AUCs than CT for tracheal invasion (0.943–0.990 vs. 0.529–0.706) and vascular invasion (0.878 for both readers). MRI achieved substantial-to-almost-perfect agreement (*κ* = 0.771–1.000), whereas CT was only moderate (*κ* = 0.369–0.487). Bootstrap analysis confirmed superior discrimination of the combined MRI score: ΔAUC + 0.19 (–0.05–0.43, *p* = 0.11) for Reader A and +0.38 (0.07–0.66, *p* = 0.02) for Reader B.

**Conclusion:**

A combined MRI T/V-score provides better accuracy and inter-reader reliability than CT for evaluating critical local invasion, even after preoperative therapy, supporting routine integration of MRI when CT findings are equivocal.

**Key Points:**

***Question*** *Determine whether a standardized MRI scoring system for tracheal and vascular invasion improves diagnostic accuracy compared with contrast‑enhanced CT in esophageal cancer.*

***Findings*** *MRI outperforms CT in detecting tracheal and vascular invasion, with higher specificity and superior inter-reader agreement using standardized scoring criteria.*

***Clinical relevance*** *Standardized MRI scoring improves staging accuracy in suspected T4 esophageal cancer, aiding surgical decision-making and helping to avoid unnecessary surgery in inoperable patients as well as incomplete (R1/R2) resections*.

**Graphical Abstract:**

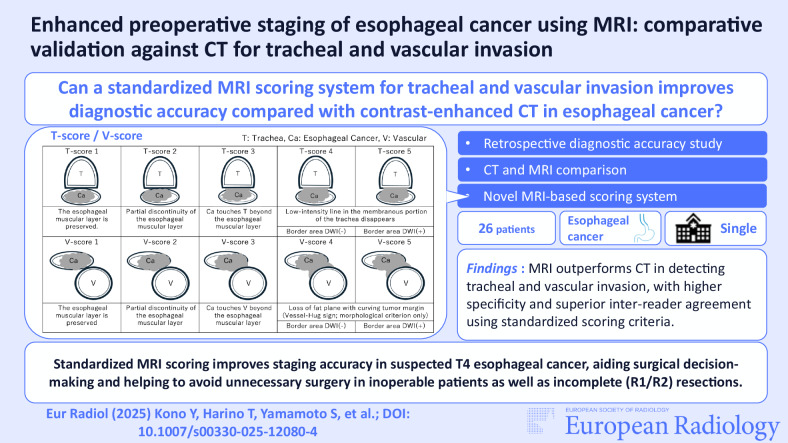

## Introduction

Esophageal cancer remains a major cause of cancer mortality worldwide [1, 2]. Once the tumor abuts or breaches the adventitia (≥ cT3), five-year survival with surgery alone falls to ≤ 25% [3, 4], and neoadjuvant chemoradiotherapy becomes standard care. Accurate identification of tracheal or aortic invasion (cT4) therefore dictates operability and treatment choice.

MRI has increasingly been explored in esophageal cancer (EC) for its ability to provide high soft-tissue contrast and functional information through diffusion-weighted imaging (DWI). Previous studies have examined MRI for T-staging and response evaluation, particularly in cervical and thoracic EC [5, 6]. However, its utility in assessing tracheal and vascular invasion—key determinants of T4b stage and surgical unresectability—remains underreported. Recent advances in MRI techniques, including high-resolution T2-weighted imaging (T2WI) and DWI, offer the potential to improve local staging precision, yet there is limited evidence validating these approaches against established modalities like contrast-enhanced CT. Recent studies have further highlighted MRI’s potential role in advanced-stage esophageal cancer, reinforcing its diagnostic value in local invasion assessment [7, 8]. Our study addresses this gap by proposing a semi-quantitative MRI scoring system for tracheal and vascular invasion and comparing its diagnostic performance with that of CT, using surgical and pathological findings as reference standards.

CT is widely used for locoregional staging but offers limited soft-tissue contrast, yielding equivocal assessments between resectable and unresectable disease [9, 10]. MRI provides superior contrast and may better delineate periesophageal structures [11–13]. Despite its advantages, MRI is not yet standard in preoperative staging due to a lack of standardized interpretation frameworks and comparative validation. We developed a five-point MRI tracheal invasion score (T-score) and vascular invasion score (V-score) and compared their diagnostic accuracy and reader agreement with CT, using pathology as reference and bootstrap AUC analysis for validation.

## Materials and methods

### Study design and patient selection

This retrospective, single-center study was approved by the Institutional Review Board of Kansai Medical University (approval number: 2024421) with a waiver for informed consent. We included 26 consecutive patients (mean age: 65 years; range: 45–78 years) with histologically confirmed esophageal cancer who underwent both CT and MRI between January 2023 and December 2024. All patients underwent contrast-enhanced CT both before and after neoadjuvant therapy, and MRI was performed only after the completion of preoperative treatment, within 14 days prior to surgery.　Inclusion criteria were: (1) histologically confirmed esophageal carcinoma, (2) availability of preoperative contrast-enhanced CT and MRI, including T2WI and DWI, and (3) subsequent surgical resection with pathological evaluation. Patients who could not undergo surgery after preoperative treatment or those with incomplete imaging data were excluded.

### Imaging protocols

MRI was acquired on Philips 1.5-T/3-T or GE 1.5-T systems (cervical lesions ungated; thoracic lesions gated) using axial T2-weighted and DWI sequences; key parameters appear in Table 1. CT acquisition followed the “12th Edition of the Japanese Classification of Esophageal Cancer” (2022). Triple-phase contrast-enhanced CT was performed (arterial, venous (35 s), and late phase (90 s)) using 100-kV tube voltage (120 kV for overweight patients). Image reconstruction was 5-mm for all phases and 2-mm thin-slice reconstruction for the arterial phase. In patients with renal dysfunction, the contrast media dose was reduced, and only venous-phase or unenhanced scans were acquired, with both 5-mm and 2-mm reconstructions used for interpretation.

**Table 1:** Standard MRI parameters for Achieva dStream 1.5 T

Image parameters	Cervical (dStream Flex S coil)		Thoracic (Torso coil)	
	T2WI	DWI	T2WI	DWI
Sequence/ETL	TSE/34	EPI/−	TSE/31−	EPI/−
ECG-synchronized/Respiration-synchronized	No/No	No/No	Yes/Yes	No/No
FOV	140 × 140	140 × 140	230 × 230	320 × 261
TR / TE /TI (ms)	2000–4000/80–90/−	4500/60–70 /183	1000–2500^a^/90–100/−	8000–10,000/60–70/183
Bandwidth (kHz)	± 31	± 51.2	± 76	± 170
NEX	3	b 0 → 3 b 1000 → 13	2	b 0 → 1 b 1000 → 8
Slice thickness/gap (mm)	3/−1	4/−1	4/−1	4/−0.4
Matrix	256 × 179	84 × 49	272 × 257	108 × 86
Imaging time	4	3	4–5	3
B-value (mm^2^/s)	-	0 and 1000	-	0 and 1000

*NEX* Number of excitations, *ETL* Echo train length, *TSE* Turbo spin echo, *EPI* Echo-planar imaging, *ECG* Electrocardiogram, *FOV* Field of view, *TR* Repetition time, *TE* Echo time, *Tl* Time inversion

^a^ TR was defined as the RR interval of 2 heartbeats in electrocardiogram synchronization

Axial images served as the primary viewing plane for all evaluations. When further assessment was necessary, multiplanar reconstructions (MPRs) were generated along planes tangential and perpendicular to the tumor–organ interface.

### Image analysis

CT Assessment: Preoperative contrast-enhanced CT images were qualitatively assessed by two radiologists with a focus on tracheal and vascular invasion signs, such as obliteration of the fat plane, organ deformity, and tumor contact angle. T-staging was categorized based on the 12th edition of the Japanese Classification of Esophageal Cancer (Japan Esophageal Society). The CT-based sub-staging criteria for cT3r, cT3br, and cT4 are summarized in Table 2. In this study, CT positivity for T4 invasion was defined as cT3br or higher, according to national treatment algorithms in which cT3br typically prompts neoadjuvant therapy.

**Table 2:** CT-based T-staging criteria in esophageal cancer

CT T-substage	Definition	Key imaging features
cT3r (resectable)	No signs of adjacent-organ invasion	Clear fat plane; Picus angle < 90°
cT3br (borderline-resectable)	Invasion cannot be excluded	Blurred or partially obliterated fat plane; Picus angle ≥ 90° for ≥ 10 mm; no definite organ wall destruction
cT4	Obvious invasion of adjacent mediastinal organs	Loss of fat plane plus deformity or narrowing of the trachea, bronchus, aorta, or other structures

**Fig. 1:**
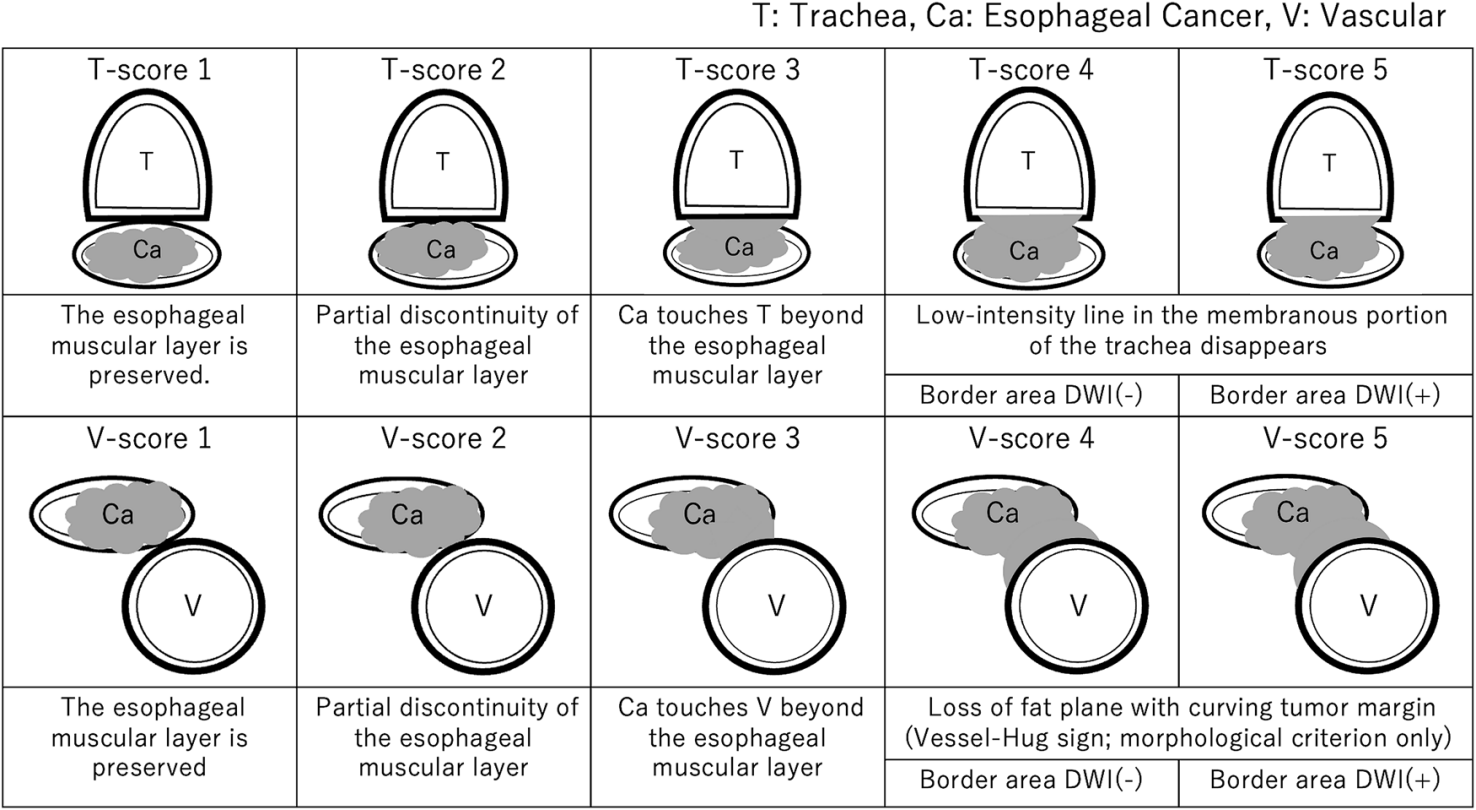
Schematic of the MRI-based T-score (tracheal invasion) and V-score (vascular invasion). Each ranges from score 1 (intact muscular layer) to score 5 (definitive invasion confirmed by a corresponding high signal on DWI). This stepwise approach facilitates consistent MRI evaluation of local tumor spread involving the trachea or major vessels

MRI Assessment: MRI-Based Scoring System; a reference schematic illustrating the diagnostic criteria for T-score and V-score on MRI is provided as Fig. 1. Two five-point scales were used to assess tracheal (T-score) and major vascular (V-score) invasion on MRI. Evaluations were primarily based on axial T2WI, and in cases suggestive of invasion (scores 4 or 5), DWI findings were reviewed to confirm or refine the interpretation.

### Tracheal invasion (T-score)

Tracheal invasion was rated on a five-point scale (T-score). A score of 1 indicated preservation of the esophageal intrinsic muscular layer. A score of 2 represented partial discontinuity of the muscular layer. A score of 3 denoted tumor extension beyond the muscularis propria with direct contact to the trachea. A score of 4 was defined as loss of the normal low-intensity line in the tracheal membranous portion on T2WI, representing the peritracheal tissue and circular smooth muscle, without a corresponding high signal in the border area on DWI. A score of 5 indicated anatomical disruption with a corresponding high signal in the border area on DWI.

### Vascular invasion (V-score: vessel-hug sign-based)

In this study, vascular invasion was assessed specifically for the thoracic aorta and the right subclavian artery. Vascular invasion was similarly evaluated on a five-point scale (V-score). A score of 1 indicated preservation of the esophageal muscular layer. A score of 2 represented partial discontinuity of that layer. A score of 3 was defined as tumor extension beyond the muscularis propria, contacting the thoracic aorta or right subclavian artery. A score of 4 was assigned when the tumor displayed a Vessel-Hug sign (VH-sign), defined morphologically as loss of the intervening fat plane with a smooth, concave tumor margin curving along the vessel wall, on T2WI, without a corresponding high signal on DWI. A score of 5 required the VH-sign plus a matching high-signal rim on DWI, indicating definite invasion.

### Inter-reader analysis

Two fellowship-trained radiologists (15 years; 6 years) independently scored images while blinded to pathology. Additionally, each radiologist was given a 4-week washout period between reading MRI and CT studies for the same patient to reduce recall bias. The readers were instructed to refrain from adjusting their initial scores after seeing subsequent images. To further minimize potential bias, both readers were not informed of any other clinical data, which was limited to image data only.

For each modality, raw score agreement was first determined by the proportion of identical assessments. Given that the MRI-based T-score and V-score each used a five-point scale, we additionally explored the impact of simplifying the data via binary cutoffs (e.g., T-score ≥ 4 or ≥ 5, V-score ≥ 5). CT-based T-staging was also categorized as negative (cT1–cT3r), equivocal (cT3br), or positive (cT4). These thresholds were chosen based on clinical relevance and preliminary analyses, aiming to reduce ambiguity in borderline cases.

### Reference Standard

Tracheal invasion was histologically confirmed in 7 cases, with one additional case diagnosed intraoperatively. Vascular invasion was histologically confirmed in 6 cases. Endoscopic ultrasound (EUS) was not performed because the study focused on advanced-stage esophageal cancer. Treatment decisions regarding T-staging were primarily based on CT findings, with MRI used as a supplementary reference.

### Statistical analysis

All data were analyzed using JMP® Student Edition 18.2.0 (SAS Institute Inc.) and Python 3.10 with scikit-learn and custom scripts. Statistical significance was set at *p* < 0.05. Sensitivity, specificity, PPV, NPV and accuracy were calculated against pathological findings; Cohen’s or weighted *κ* assessed inter-reader agreement. Cases with missing T- or V-scores for either reader were excluded list-wise from the corresponding inter-observer and diagnostic‐accuracy analyses. ROC AUCs were compared between the combined MRI score (max(T, V)) and the CT T-stage. Because CT scores are ordinal with zero variance under DeLong, patient-level bootstrap resampling (2000 iterations) provided ΔAUC, 95% percentile confidence interval (CI) and empirical two-sided *p*. Missing values for T- or V-scores were rare (< 2%) and handled by list-wise exclusion from the corresponding analyses.

## Results

### Patient characteristics

The study included 26 patients (17 men and 9 women; mean age, 65 years (range, 39–82 years)) with pathologically confirmed esophageal cancer. Of these 26 patients, 22 received chemotherapy alone as preoperative treatment, 3 received chemoradiotherapy, and 1 received radiotherapy alone. The tumor was located in the cervical esophagus in 5 patients, the upper esophagus in 1 patient, the middle esophagus in 16 patients, and the lower esophagus in 4 patients. In 22 cases, the possibility of tracheal invasion required evaluation, whereas in 19 cases, the possibility of invasion into the thoracic aorta or right subclavian artery was assessed. For these cases, T and V-scores were used to determine the extent of local invasion. Among the 26 patients, tracheal invasion was confirmed in 7 cases (1 cervical, 6 mid-thoracic) and vascular invasion in 6 cases (5 thoracic aorta, 1 right subclavian artery). Although cT4b is generally considered inoperable, conversion surgery was performed in selected patients when R0 resection was deemed feasible after neoadjuvant therapy. However, pathological evaluation revealed R1 resection in 3 cases and R2 in 6 cases, indicating the challenges of achieving curative margins in this cohort. Tumor regression grade (TRG) was evaluated in all resected specimens according to the Japanese Classification of Esophageal Cancer (12th edition). Among the 26 patients, 1case achieved Grade 3 (no residual tumor), 5 cases had Grade 2 (≥ two-thirds tumor necrosis), 5 cases had Grade 1b (≥ one-third but < two-thirds necrosis), and 15 cases had Grade 1a or 0 (< one-third or no response), indicating a wide variation in pathological response.

### Diagnostic performance of MRI and CT

**Fig. 2:**
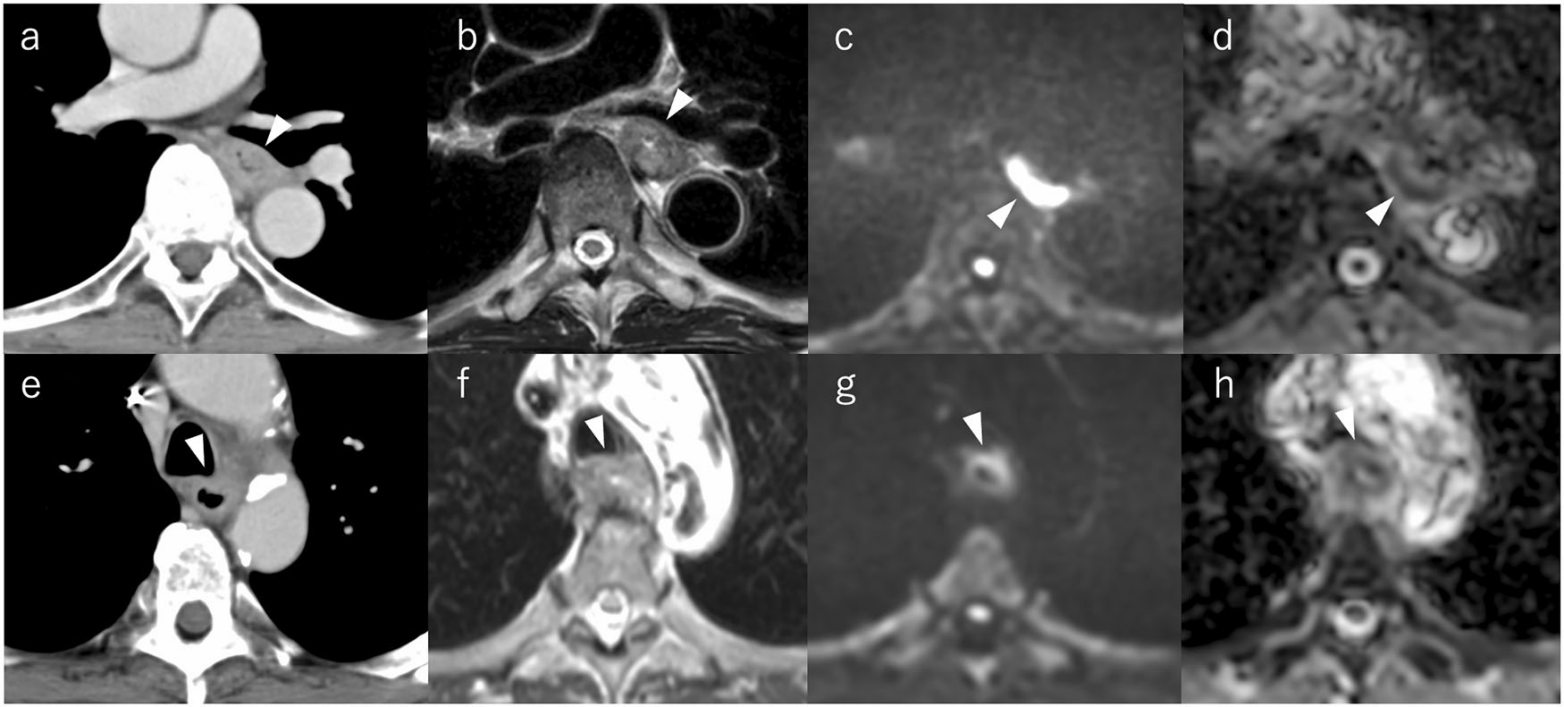
Representative cases of tracheal invasion. Upper row: The contrast-enhanced CT (**a**) shows compression of the left main bronchus and narrowing of the lumen due to the esophageal tumor (readers: cT4 vs. cT3br), whereas in the MRI T2WI (**b**), although the esophageal muscular layer is partly unclear, the low-density line of the left main bronchus is maintained, and in DWI (**c**) and ADCmap (**d**), the localized decrease in diffusion is limited to the posterior wall of the esophagus, and it was judged that there was no obvious infiltration (both readers: T-score 3). Lower row: Contrast-enhanced CT (**e**) suggests slight bronchial irregularity (readers: cT3br vs. cT3r), but MRI T2WI (**f**) and DWI (**g**), ADCmap (**h**) demonstrate loss of the bronchial wall’s low-intensity line (both readers: T-score 5). Pathology confirmed pT4

**Fig. 3:**
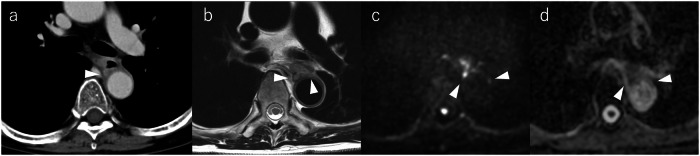
Representative case of major vascular invasion. Contrast-enhanced CT (**a**) shows a posteriorly protruding tumor with a Picus angle < 90°, (readers: cT3r vs. cT3br). MRI reveals a “VH-sign” on T2WI (**b**) plus a high signal on DWI (**c**), a decrease in apparent diffusion coefficient (**d**) was observed at the aortic interface (both readers: V-score 5). Pathology confirmed T4 vascular invasion

MRI outperformed CT for tracheal invasion (AUC 0.943–0.990 vs. 0.529–0.706) and vascular invasion (AUC 0.878 vs. ≤ 0.706; Table 3). Representative case comparisons are shown in Figs. 2 and 3. The addition of DWI had variable effects. Higher T-score and V-score thresholds that required DWI positivity improved specificity but also introduced potential disagreement in borderline cases, reflecting the difficulty of consistently interpreting DWI signals in thoracic imaging. Optimal cutoffs for MRI-based scores were ≥ 4 (Reader A) and ≥ 5 (Reader B) for tracheal invasion, and ≥ 5 for vascular invasion in both readers. For CT-based pT4 classification, ≥ cT3br improved sensitivity but decreased specificity compared with using cT4 alone. Using combined MRI criteria, specificity increased to ≥ 0.88 without loss of sensitivity (Table 4).

**Table 3:** Diagnostic performance of MRI and CT

	MRI-T		MRI-V		CT	
	Reader A^a^	Reader B^a^	Reader A^a^	Reader B^a^	Reader A^a^	Reader B^a^
Sensitivity	1	0.87	0.83	0.83	1	0.83
Specificity	0.87	0.87	1	1	0.40	0.50
PPV	0.78	0.75	1	1	0.33	0.33
NPV	1	0.93	0.93	0.93	1	0.91
Accuracy	0.91	0.86	0.95	0.95	0.54	0.58

*PPV* Positive predictive value, *NPV* Negative predictive value

^a^ Cutoff values determined by Youden Index: MRI-T (Reader A) = 4, MRI-T (Reader B) = 5, MRI-V (Reader A/B) = 5, CT(Reader A/B) = T3br

**Table 4:** Diagnostic performance for pT4 prediction (MRI: T or V positive).

Metric	Reader A		Reader B	
	MRI^a^	CT^b^	MRI^a^	CT^b^
Sensitivity	0.889	1.000	0.889	0.667
Specificity	0.882	0.471	1.000	0.471
PPV	0.800	0.500	1.000	0.400
NPV	0.938	1.000	0.944	0.727
Accuracy	0.885	0.654	0.962	0.538

*PPV* Positive predictive value, *NPV* Negative predictive value

^a^ For combined MRI assessment, pT4 was diagnosed when either tracheal invasion (T-score) or vascular invasion (V-score) exceeded the predefined threshold (≥ 4 for Reader A, ≥ 5 for Reader B)

^b^ CT-based pT4 prediction was defined as a score of ≥ T3br for both readers. Diagnostic performance was compared using McNemar’s test for paired proportions.

### Inter-reader agreement

The proportion of identical raw scores between readers was 68.2% for T-score, 68.4% for V-score, and 57.7% for CT T-stage. Using T-score ≥ 5 rather than ≥ 4 improved the kappa from 0.436 (moderate) to 0.771 (substantial), indicating that requiring both anatomic disruption on T2WI and a corresponding high DWI signal reduced subjective variability. V-score ≥ 5 achieved perfect agreement (*κ* = 1.00), and T-score ≥ 5 substantial agreement (*κ* = 0.77), whereas CT staging remained moderate (*κ* ≤ 0.49; Table 5).

**Table 5:** Inter-reader agreement for MRI- and CT-based scoring systems

Score type	Classification type	Agreement metric	*κ* coefficient	Raw agreement (%)
MRI T-score	Raw score (1–5)	Weighted *κ*	0.823	68.2
MRI T-score	Binary (≥ 4)	Cohen’s *κ*	0.436	72.7
MRI T-score	Binary (≥ 5)	Cohen’s *κ*	0.771	81.8
MRI V-score	Raw score (1–5)	Weighted *κ*	0.704	68.4
MRI V-score	Binary (≥ 5)	Cohen’s *κ*	1.000	100.0
CT T-stage	Trichotomous (−/±/+)	Weighted *κ*	0.487	57.7
CT T-stage	Binary (≥ T3br)	Cohen’s *κ*	0.369	80.8

Cohen’s *κ* was used to assess inter-reader agreement for binary classification tasks. Weighted *κ* was applied to ordinal data including raw scores (1–5) and trichotomous CT staging. “Trichotomous (−/±/+)” refers to three categories of CT-based T-staging: negative (T1–T3r), equivocal (T3br), and positive (T4)

When combining T-score and V-score criteria (MRI positive if either is positive), MRI demonstrated significantly higher specificity than CT for both readers (*p* = 0.016 for Reader A, *p* = 0.004 for Reader B), while differences in sensitivity were not statistically significant (*p* = 1.000 and *p* = 0.625, respectively). This suggests that MRI-based combined scoring offers a more specific alternative to CT without compromising sensitivity for predicting pT4 disease.

### Combined MRI vs. CT: AUC comparison

Bootstrap analysis confirmed higher discrimination for MRI (ΔAUC + 0.19 [CI –0.05–0.43], *p* = 0.11 for Reader A; +0.38 [0.07–0.66], *p* = 0.02 for Reader B; Supplementary Table 1).

## Discussion

Our results confirm that MRI—with standardized T- and V-scores—outperforms CT for both tracheal and vascular invasion. AUCs reached 0.94–0.99 (T-score) and 0.88 (V-score) vs. ≤ 0.71 for CT, while *κ* rose from «moderate» to «substantial–almost-perfect». These gains were largely driven by high-resolution T2WI and anatomically standardized scoring. DWI was incorporated as a secondary tool to assist in tracheal assessment. Although its reliability here was limited by treatment-related changes (e.g., fibrosis), magnetic susceptibility, and lack of respiratory or cardiac gating, recent work has suggested that multiparametric MRI, including DWI, may help assess treatment response in esophageal cancer [14]. In particular, Chapellier et al [12] demonstrated that DWI, as part of multiparametric MRI, can aid in assessing treatment response after neoadjuvant therapy for esophageal cancer by helping to distinguish viable tumor from fibrosis. While our study focused on anatomical invasion, their findings highlight the complementary value of functional imaging.

Another factor contributing to the differing performance between MRI and CT may be the reliance on the Picus angle in CT interpretation. This angle—formed between the esophageal wall and adjacent great vessels—serves as an indirect surrogate for vascular invasion. However, it is highly dependent on the imaging plane and may be confounded by surrounding anatomical distortion, especially after neoadjuvant therapy. In contrast, MRI enables direct visualization of tissue planes and vessel wall infiltration, eliminating the need for angle-based inference. Thus, the MRI-based V-score employed in this study focused solely on anatomical criteria, such as the VH-sign and DWI positivity, which may offer a more reproducible assessment of vessel invasion across readers.

When using a combined scoring approach, defining MRI positivity as either tracheal (T-score) or vascular (V-score) invasion, the diagnostic accuracy of MRI improved markedly. MRI achieved higher specificity than CT for both readers (*p* = 0.016 for Reader A, *p* = 0.004 for Reader B), while maintaining high sensitivity (0.889). These findings suggest that combined MRI scoring may be a reliable strategy for identifying pT4 disease, potentially offering a more balanced diagnostic profile than CT. A notable strength of this study is that all patients underwent surgical resection with pathological assessment, providing a robust gold standard for evaluating imaging accuracy. Furthermore, we proposed and validated a standardized MRI scoring system with high inter-reader reproducibility, which may facilitate more consistent and objective evaluation of local tumor invasion in clinical practice. Despite some limitations, such as the single-center design, small sample size, and the use of multiple scanner types, our results strongly suggest that MRI can complement or even surpass CT in evaluating local esophageal tumor invasion.

The bootstrap analysis confirmed that, even after accounting for the coarse and ordinal nature of CT staging, the combined MRI score retains superior discriminatory power. Although Reader A did not reach statistical significance, the trend toward higher AUC (+ 0.19) and the significant advantage observed for Reader B (+ 0.38, *p* = 0.02) support the robustness of the MRI criteria. The discrepancy between readers likely reflects differences in confidence when interpreting borderline CT findings (T3br). Importantly, this reader-dependent variability disappeared with MRI, underscoring the value of standardized T- and V-scores.

Assessing tracheal and vascular invasion on CT is inherently challenging due to limited soft-tissue contrast, especially when the fat plane is compressed or absent [15, 16]. Additionally, the anatomical proximity of the esophagus to adjacent mediastinal structures can result in equivocal findings, particularly after neoadjuvant therapy when fibrosis or edema may mimic invasion [17]. Some studies have proposed that preparation with oral contrast or gas insufflation might improve delineation between the esophageal wall and surrounding tissues on CT [15, 18]; however, such techniques are not routinely employed in preoperative staging and remain limited in resolving ambiguous cases. EUS is useful in T-staging, especially for assessing submucosal and muscular extension, but it is not suitable for assessing mediastinal structures, especially vascular lesions, due to the narrow field of view and procedural difficulties in advanced cancer [19], and was not performed in the patients in this study.

These results underscore the potential of standardized MRI scoring as a clinically useful alternative to EUS or CT in the preoperative evaluation of locally advanced esophageal cancer. Given the promising results of this study, a prospective clinical trial is ongoing at our institution to further evaluate the utility of MRI-based T- and V-scoring. Its incorporation into clinical practice is under consideration, contingent upon further validation of its reproducibility and overall clinical benefit.

## Limitations and future directions

Limitations include the single-center design, small cohort, and multi-vendor MRI. A multicenter prospective study with harmonized protocols is needed before routine adoption.

## Conclusion

MRI, utilizing standardized T- and V-scores, demonstrated superior diagnostic accuracy and inter-reader agreement compared to CT in assessing tracheal and vascular invasion in esophageal cancer. These findings support the integration of MRI into preoperative staging protocols for patients with suspected T4 disease.

## Supplementary information

ELECTRONIC SUPPLEMENTARY MATERIAL

## Data Availability

Custom Python scripts used for bootstrap analysis are available from the corresponding author on reasonable request.

## Abbreviations

AUC: Area under the receiver‑operating‑characteristic curve
DWI: Diffusion-weighted imaging
NPV: Negative predictive value
PPV: Positive predictive value
ROC: Receiver-operating-characteristic
T-score: Tracheal invasion score
V-score: Vascular invasion score
*κ*: Cohen’s kappa
