# Supplementary material for: MRI outperforms CT for tracheal and vascular invasion staging in esophageal cancer
Source: Eur Radiol. 2025 Oct 25;36(4):2842–50. doi: 10.1007/s00330-025-12080-4 (PMC13035738; doi:10.1007/s00330-025-12080-4)
Supplement: Supplementary file 1 — ELECTRONIC SUPPLEMENTARY MATERIAL [file 330_2025_12080_MOESM1_ESM.pdf]

# MRI Outperforms CT for Tracheal and Vascular Invasion Staging in Esophageal Cancer

## ELECTRONIC SUPPLEMENTARY MATERIAL

Supplementary table 1. Bootstrap comparison of AUCs between the combined MRI score and CT T-stage (12th Japanese Classification; CT positive = ≥ cT3br). CI = confidence interval, $\Delta AUC = AUC_{MRI} - AUC_{CT}$.

| Reader | $AUC_{MRI}$ | $AUC_{CT}$ | ΔAUC | 95 % CI | p |
|---|---|---|---|---|---|
| A | 0.899 | 0.706 | +0.193 | −0.046 to 0.427 | 0.110 |
| B | 0.905 | 0.529 | +0.376 | 0.065 to 0.658 | 0.021[†] |

CI : confidence interval, ΔAUC : difference in AUCs.
